# The Human Transcriptome: An Unfinished Story 

**DOI:** 10.3390/genes3030344

**Published:** 2012-06-29

**Authors:** Mihaela Pertea

**Affiliations:** McKusick-Nathans Institute of Genetic Medicine, Johns Hopkins University School of Medicine, Baltimore, MD 21205, USA; E-Mail: mpertea@jhu.edu; Tel.: +1-443-287-0972

**Keywords:** transcriptome, pervasive transcription, RNA-seq, mRNA, ncRNA

## Abstract

Despite recent technological advances, the study of the human transcriptome is still in its early stages. Here we provide an overview of the complex human transcriptomic landscape, present the bioinformatics challenges posed by the vast quantities of transcriptomic data, and discuss some of the studies that have tried to determine how much of the human genome is transcribed. Recent evidence has suggested that more than 90% of the human genome is transcribed into RNA. However, this view has been strongly contested by groups of scientists who argued that many of the observed transcripts are simply the result of transcriptional noise. In this review, we conclude that the full extent of transcription remains an open question that will not be fully addressed until we decipher the complete range and biological diversity of the transcribed genomic sequences.

## 1. Background and Introduction

The transcriptome of a cell is the collection of all the RNA molecules, or transcripts, present in that cell. To generate the transcriptome, the DNA of an organism is first transcribed by RNA polymerase to create complementary RNA strands, which in turn are spliced to remove introns, producing mature transcripts that contain only exons. For many years, it was assumed that these RNA transcripts were primarily used as templates for translation to proteins. The vast majority of the remaining human genome, which is not protein coding, was thought to be non-functional and therefore considered “junk” DNA [1]. Soon after the publication of the human genome sequence in 2001 [2,3], a new view emerged, holding that only a small percentage of the human transcriptome is clearly translated into proteins [4,5,6], and most of the remaining transcripts have unknown purposes. In recent years, the number and variety of known RNA genes has grown dramatically, and in addition to protein-coding messenger RNAs (mRNAs), the catalog of transcribed elements now includes a myriad of non-coding RNAs (ncRNAs) that play multiple structural and regulatory roles in the molecular biology of the cell [7]. 

Ever since the discovery of the genetic code, scientists have labored to decipher the complete human transcriptome. It was only with the emergence of automated DNA sequencing in the 1980s that real progress was made in this direction [8]. In the 1990s, scientists realized the value of using expressed sequence tag (EST) sequencing to rapidly identify expressed genes, or at least fragments of those genes, in many human tissues [9,10]. Although at the time EST sequencing was considered a very high-throughput technique, both costs and technical limitations prevented it from producing a complete transcript catalog. As a consequence, much of our knowledge of the protein-coding portion of the human transcriptome relied on different computational gene prediction methods [11,12]. 

Various other technologies were developed to complement the traditional EST approach. These include tag-based methods such as serial analysis of gene expression (SAGE) [13], cap analysis of gene expression (CAGE) [14], and massively parallel signature sequencing (MPSS) [15]. Unlike the EST approach, the tag methods uniquely identify each transcript to achieve gene-level expression quantification. However they are generally unable to distinguish specific isoforms. In addition, most of them are based on traditional Sanger sequencing technology, making them very expensive to apply on a large scale. 

Hybridization-based microarrays provided the first relatively inexpensive way to detect and quantify transcripts on a large scale [16,17,18]. These include transcription tiling arrays, which allow the mapping of transcribed regions to a very high resolution, from 5 to 50 base pairs (bp), depending on probe density [19,20]. They have several advantages over previous methods, including their high throughput and their ability, with some designs, to quantify distinct spliced isoforms [21]. However, because of differences in hybridization strength, cross-hybridization, and other experimental variables, microarrays provide a noisy output signal. In addition, they can only measure genes for which the sequence and the precise exon-intron boundaries are known, making them unable to identify novel genes or novel splicing events [22,23]. 

Recently, RNA-seq methods technologies provide unprecedented opportunities for characterizing the set of RNA transcripts produced in a cell [24,25,26,27,28]. Called a “revolutionary tool for transcriptomics”, RNA-seq is the first sequencing-based method that allows the entire transcriptome to be surveyed in a very high-throughput and quantitative manner [29]. Unlike hybridization-based methods, it is not limited to the detection of known transcripts, and it can measure a much larger range of expression levels. Among its other advantages, RNA-seq data has relatively low background noise; it achieves base-pair resolution, allowing precise identification of exon and intron boundaries; and it can detect single nucleotide polymorphisms (SNPs) and other variants within transcripts. Although RNA-seq has already dramatically changed the landscape of genetic studies, it is clear that many years remain before we will have a complete catalogue of human genes and their expressed isoforms. 

## 2. The Diversity of the Transcriptome

### 2.1. Various Classes of ncRNAs

Over the past decade, many studies have revealed an unexpected level of diversity in the human transcriptome, which in turn has required scientists to expand their definition of a gene. The traditional definition of a gene—a DNA sequence that is transcribed to produce a functional product—has been expanded to include not only to the ~22,000 protein-coding genes present in the human genome [11], but also a myriad of non-protein coding sequences. These set of transcribed non-protein coding DNA sequences show complex patterns of expression and regulation [30], and they are no longer restricted to the well known ribosomal and transfer RNAs (rRNAs and tRNAs, respectively). Furthermore, when we introduce these new and growing functional RNAs into our gene counts, the number of genes in the human genome increases from ~22,000 (which includes only protein-coding genes) to the 2001 estimates of about 30,000–40,000 genes [31]. 

The discoveries of endogenous small interfering RNA (siRNA) [32] and microRNA (miRNA) [33] genes represented dramatic breakthroughs in our understanding of the transcriptome. These two classes of small ncRNAs play a central role in RNA interference by binding to specific mRNA molecules to either increase or decrease their activity. Various other classes of ncRNAs have a now-broadly recognized functional role. These include regulatory RNAs such as PIWI-interacting RNAs (piRNAs), promoter-associated RNAs (PARs), transcription initiation RNAs (tiRNAs), X-inactivation RNAs (xiRNAs), and many others [34,35]. Among them, the long non-coding RNAs (lncRNAs), defined as ncRNAs longer than 200 bp, are probably the least well-understood transcripts. Although few of them have been experimentally studied, a view is emerging that these are key regulators of epigenetic gene regulation in mammalian cells [36]. 

Large intergenic RNAs (lincRNAs) are a subclass of lncRNAs that do not overlap protein-coding regions. Cabili *et al*. [37] catalogued more than 8,000 lincRNAs (58% of which were novel) using an integrative approach that unifies existing annotation sources with transcripts assembled from RNA-seq data collected from 24 tissues and cell types. Several global properties of lincRNAs were evidenced by this study:

- they are expressed in a highly tissue-specific manner compared to protein-coding genes,
- they are typically co-expressed with their neighboring genes, and
- they only show moderate conservation in other species.

The functional classification of lincRNAs is far from complete, even though Cabili *et al*. assigned putative functions to many predicted lincRNAs based on the functions of protein-coding genes with similar expression patterns. 

### 2.2. Alternative Splicing

Even when considering only protein-coding RNAs, the scientific community still does not have a complete picture of the transcriptome. Not only is there uncertainty about the exact number of human protein-coding genes, but recent evidence has emerged to show that different humans have slightly different individual gene sets [38,39,40]. The number of mature mRNA transcripts is even less certain, and varies across tissues and different stages during cell differentiation [41,42]. Further complicating matters, we now know that more than 90% of multi-exon protein-coding genes undergo alternative splicing [43,44], which is considered to play a major role in increasing cellular and functional diversity in the transcriptomes of higher eukaryotes [45]. However, we do not yet know the function of the vast majority of alternatively spliced human transcripts, and it is now clear that alternative splicing does not simply act to generate variant protein sequences [46].

Alternative splicing also affects ncRNA genes, about 30% of which produce at least one alternatively spliced transcript [47]. Cabili *et al*. found that lincRNAs, although shorter and with fewer exons than mRNAs, are also alternatively spliced with an average of 2.3 isoforms per locus [37]. New transcripts are continuously being discovered [19,41,48,49], strengthening the observation that we are far from determining all transcript isoforms. 

### 2.3. Estimating the Annotated Human Transcript Count

In an attempt to identify how many human transcripts are currently annotated, I combined all human gene annotations from Ensembl (release 64) [50], NCBI’s RefSeq database [51], and the UCSC Genome Browser [52] with the lincRNAs catalogued by Cabili *et al*. [37]. After eliminating redundant transcripts (*i.e.*, transcripts with identical annotation as an already included transcript from one of the databases), I divided the remaining ones into three categories: mRNAs if they were annotated as protein-coding transcripts, long ncRNAs if they were annotated as non-coding and were at least 200 bp long, and small ncRNAs otherwise. 

**Figure 1:**
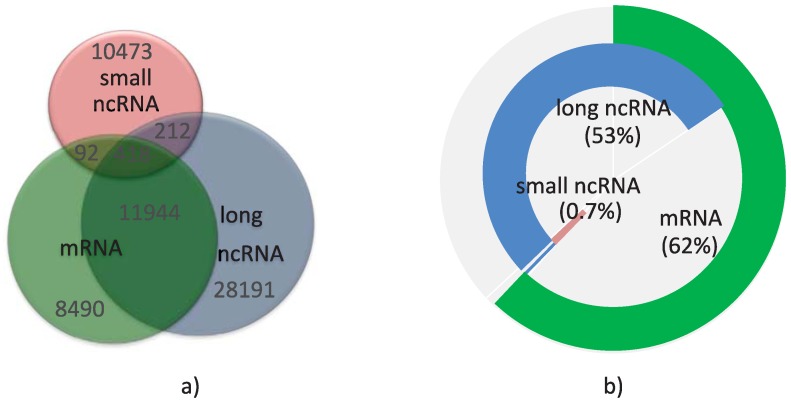
Composition of the human transcriptome. (**a**) Venn diagram of the number of loci containing mRNA transcripts (green), long ncRNAs (blue), and small ncRNAs (red); (**b**) Base pair coverage of the transcriptome by the three categories of transcripts.

As also observed by others [53], I found a highly complex architecture in the human transcriptome, in which some base pairs could be part of many overlapping transcripts in any of the three categories, and emanating from both strands of the genome. Loci containing all three categories of transcripts were not frequent (see Figure 1a). Not surprisingly, the annotations include more mRNA than ncRNA transcripts, possibly due to a bias towards annotating protein-coding transcripts, although loci with at least one ncRNA are more numerous than loci containing one or more mRNAs (see Table 1). Overall, annotated transcripts today cover 4.62% or 3.85% of the human genome, depending on whether or not we include pseudogenes. Expression of pseudogenes is controversial, with some reports suggesting that they might be transcribed and could play a significant part in gene regulation [54,55]. They cover about 30% of the total base pairs included in all ncRNA transcripts. Figure 1b shows the base pair coverage of the human transcriptome (including pseudogenes) by the three categories of transcripts. I found that 62% of the base pairs in the transcriptome are part of mRNAs, supporting the fact that ncRNAs tend to be smaller in length than mRNAs. 

**Table 1:** Number of known annotated transcripts and human gene loci collected from Ensembl, NCBI’s RefSeq, UCSC Genome Browser, and Cabili *et al*.’s lincRNA catalog. A single locus typically contains multiple transcripts, particularly for mRNAs.

Annotation	mRNA	Long ncRNA	Small ncRNA
Transcripts	111,451	89,981	11,366
Loci	20,944	40,765	11,195

### 2.4. RNA Editing

RNA editing is another cellular process that contributes to the complex landscape of mammalian transcriptomes. In the RNA editing process, single nucleotide changes occur after DNA has been transcribed into RNA. The resulting RNA transcripts may produce altered proteins, or they may disrupt translation more severely [56]. Two RNA editing mechanisms are known in humans, causing two types of substitutions: adenosine to inosine, and cytosine to uracil. The A-to-I editing, also called A-to-G, is a process mediated by a family of adenosine deaminases (ADARs) that act on RNA and replace certain adenosines (A) with inosines, which then act as guanosines (G) during translation [57,58]. Similarly, the C-to-U switches are mediated by APOBEC1 [59,60,61]. 

Until recently considered a rare event, RNA editing is now believed to affect both coding and non-coding sequences of thousands of genes, including ncRNAs [56,62,63]. A 2011 study by Li *et al*. [64] looked at RNA-seq and DNA sequence data from 27 individuals and reported that RNA-DNA differences (RDDs) are not limited to the two previous types of substitutions described above. In their study, Li *et al*., observed all 12 possible RNA-DNA substitutions at more than 10,000 exonic sites, most of them present in multiple individuals and in different cell types. Their result suggests that previously unknown RNA editing mechanisms may be active in humans. However, this result has been strongly contested by several other groups, who argued that the vast majority of the observed RDDs were technical artifacts, mostly due to read mapping errors or systematic sequencing errors [65,66,67,68]. Nevertheless, RNA editing has an important role in molecular biology, and recent studies show that it may produce even more transcriptome diversity than alternative splicing [69]. 

## 3. Reconstructing the Transcriptome

As discussed above, high-throughput RNA sequencing surpasses all previous technologies in its ability to profile the extent and complexity of eukaryotic transcriptomes. The latest generation of sequencing machines can generate up to 600 gigabases (Gb) in a single run, equivalent to 200-fold coverage of the human genome. The 600 Gb is produced in the form of 6 billion short reads, each approximately 100 bp in length (using the Illumina HiSeq sequencer), and assembling these reads into chromosomes is a very complex, highly specialized task. Therefore one of the main challenges posed by RNA-seq is a computational one. Here I will briefly mention some of the most common bioinformatics systems for transcriptome assembly, and the challenges faced by these systems. For a more comprehensive review of next-generation transcriptome assembly methods, the interested reader can consult several recent reviews [70,71,72].

Although many programs have been developed for whole-genome assembly (e.g., [73,74,75]), these methods cannot be directly applied to transcriptome assembly due to specific characteristics of RNA-seq data sets. Genome assembly programs assume that the DNA sequence’s depth of coverage is relatively uniform across the genome. This is not true for transcripts, which have highly variable sequence coverage depending on their expression levels. Sequence depth is used to indicate repeats by genome assemblers, which are designed to take this into account. Another confounding fact for genome assemblers is that alternative transcripts from the same locus typically share exons that are difficult to assemble unambiguously. Specific features of RNA-seq data (e.g., strand-specific sequencing or partially covered gene transcripts from low-abundance genes [48]) can also confound a whole-genome assembly algorithm. Therefore new methods have had to be developed to address the particular characteristics of transcriptome assembly.

There are two main approaches for assembly of a transcriptome: a genome-guided approach when a reference genome is available; or *de novo* assembly, which does not need a genome reference and can theoretically reconstruct transcripts that are transcribed even from parts missing from that genome’s assembly. *De novo* transcriptome assembly is far more challenging in higher eukaryotes due to the large number of genes, the great variation in their expression levels, and especially because of the large number of alternatively spliced transcript variants. For this reason, *de novo* methods are primarily used for organisms that lack a sequenced reference genome.

Read mapping is one of the main technical challenges of genome-guided approaches. Alignment of short reads to the reference genome is a challenge in itself, but with RNA-seq data these reads may be sequenced from exons and exon-exon junction regions. Methods such as Bowtie [76] and BWA [77] can be used for the alignment of reads to either a reference genome or directly to the transcriptome, but this strategy will miss novel exons and novel splicing events. Spliced aligners were developed to overcome these limitations. Some of them (e.g., TopHat [78], SpliceMap [79], MapSplice [80]) use an ‘exon-first’ approach where reads are first mapped to the genome, and then the unmapped reads are split into shorter segments and aligned independently. Other spliced aligners, such as GSNAP [81] or BLAT [82], use a ‘seed-and-extend’ strategy in which the reads are first divided into small segments (seeds) that are individually aligned to the genome, and then candidate regions are locally aligned to obtain the final spliced alignment of the read. There are different advantages to these strategies, but in general ‘exon-first’ aligners are usually faster, while ‘seed-and-extend’ ones may be slightly more sensitive by reducing the bias towards unspliced alignments in the exon-first approach.

After mapping all reads to the reference genome, transcriptome assemblers cluster the overlapping reads at each locus and build a connectivity graph representing all possible isoforms. Different transcriptome assembly programs, such as Cufflinks [41], Scripture [83], IsoInfer [84], and IsoLasso [85], use different criteria to parse the connectivity graph. Cufflinks uses a parsimony principle to generate the minimal number of transcripts that will explain all reads in the graph. If there are multiple ways to assemble a minimal number of transcripts, Cufflinks uses the read coverage across each path to decide which combination is most likely to originate from the same RNA transcript. Scripture reconstructs all possible isoforms by enumerating all possible paths in the connectivity graph that have statistically significant read coverage. While Cufflinks and Scripture estimate the abundance of transcripts after they are assembled, IsoInfer and IsoLasso assemble transcripts at the same time that they estimate their expression levels. They take two different approaches: IsoInfer uses a heuristic approach to reduce the huge search space of all valid isoforms, while IsoLasso uses a multivariate regression method that also minimizes the number of predicted transcripts. 

*De novo* transcriptome assembly methods, generally based on de Brujin graphs, are less efficient and less sensitive than genome-guided methods for the human genome. Despite that, running a *de novo* assembler in addition to a genome-guided method may produce a more comprehensive transcriptome. Because *de novo* assemblers do not need a reference genome, they can identify genes that are missing from the reference genome, such as trans-spliced transcripts and similar transcripts originating from chromosomal rearrangements. Trinity [86], Oases [87], SOAPdenovo [88], and Trans-ABySS [89] are some of the programs used for *de novo* transcriptome assembly. A recent comparative study [90] evaluated the performance of different *de novo* transcriptome assembly programs and found that Trinity performed well across various conditions, but took the longest running time; Oases consumed the most memory; SOAPdenovo required the shortest runtime but performed poorly at reconstructing full-length transcripts; and Trans-ABySS showed a good balance between resource usage and quality of assemblies. Although it would undoubtedly prove useful, there is no automated software pipeline to carry out a combined assembly strategy to bring together the high sensitivity of genome-guided assemblers with the ability of *de novo* methods to detect novel and trans-spliced transcripts.

## 4. The Size of the Transcriptome

**Figure 2:**
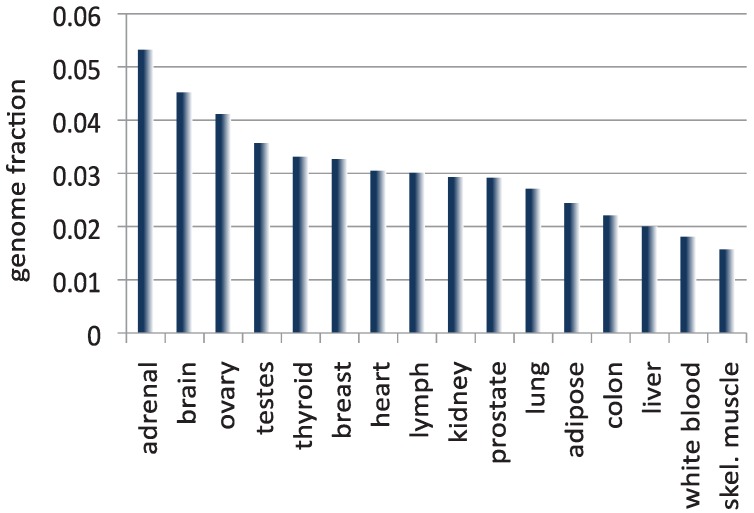
The size of the transcriptome, computed as the fraction of the total number of base pairs in the human genome covered by the assembled transcripts, for 16 normal human tissues included in the Illumina Body Map [98]. Each RNA-seq data set was mapped to the genome with TopHat [78] and assembled with Cufflinks [41]. Note that except for adrenal tissue, in which transcripts cover 5.3% of the human genome, all other reconstructed transcriptomes are smaller in size than the currently annotated transcriptome.

Less than 2% of the human genome codes for proteins [91]. As described above, if we add to this fraction the DNA sequences that correspond to annotated ncRNAs, we are still left with less than 5% of the human genome covered by known transcripts. Other reports have found that only ~5–10% of the genome is stably transcribed in cell lines [19,20,92]. My own independent analysis (Figure 2) shows that it is rare to see more than 5% of the total base pairs in the genome covered by assembled transcripts in normal human tissue. While these studies don’t capture the expression of the transcriptome at all stages in the cell development, they suggest that only a small portion of the human genome is transcribed. And yet a mounting number of studies suggest that the vast majority of the genome is transcribed at some time or other. Beginning in the early 2000s, full length cDNAs from various mouse tissues at different developmental stages, and genome-wide tiling arrays in different human tissues and cell lines revealed that much more of the mammalian genomes is transcribed than what is annotated in public databases [5,19,20,49,93,94,95]. These studies culminated with the publication in 2007 of the results from the pilot phase of the ENCODE Project [96], which estimated that as much as 93% of the human genome is transcribed in at least one cell type. Does this broad pattern of transcription mean simply that the cell creates a great deal of transcriptional noise by RNA polymerase binding accidentally (or randomly) to many sites in the genome? Or does this result challenge the long-standing view that most of the human genome is not biologically active? Scientists have conflicting opinions on the answer to this question. 

A recent study published by van Bakel *et al*. [97] claims that most ‘dark matter’ transcripts—defined as ncRNAs of unknown function - are associated with known genes. In this paper, van Bakel *et al*. argue that there is a high false-positive rate associated with the tiling array technology that was the basis of most analyses that suggested the pervasiveness of transcription. When compared to RNA-seq data, tiling arrays produce a larger proportion of low-abundance transcripts originating from intergenic and intronic regions, although tiling arrays and RNA-seq data generally agree on the location of the greatest transcript “mass.” The low coverage of intronic transcripts suggests that they might in fact represent random sampling from partially processed or unprocessed RNAs. Supporting this idea is also the observation that the transcription mass in intergenic regions increases at much lower rates than in intronic regions as the number of reads is increased. Van Bakel *et al*., also identified several thousand small transcripts that map outside known genes, however most of them could be explained as accidental by-products of enhancer activity. Overall, the authors conclude that most of the genome is not appreciably transcribed, and the majority of intergenic and intronic transcripts observed in previous studies may be attributed to biological and/or technical background noise.

Clark *et al*. [99] acknowledge that indeed most dark matter transcripts are associated with known genes, but they strongly disagree with van Bakel *et al*.’s conclusion that the genome is not as pervasively transcribed as previously reported. In their study, Clark *et al*., argue that we cannot dismiss the observations from multiple independent techniques, including RT-PCR, RACE, and Northern blot analyses, which together validated more than 90% of the identified transcripts [100,101]. They also argue that van Bakel *et al*.’s RNA-seq data suffers from insufficient sequencing depth and poor assembly, and is biased towards polyadenylated RNA, which selectively omits significant amounts of RNA as has been shown earlier [102]. Overall, similarly to other studies [103,104], Clark *et al*. find that the detection accuracy of tiling arrays is not significantly lower than that of RNA-seq, and they conclude that a significant fraction of dark matter RNA comes from very long, intergenic transcribed regions. 

In a subsequent paper [105], van Bakel *et al*. agree with the fact that most of the genome appears to be transcribed. But given the various sources of extraneous reads, both biological and laboratory-derived, they expect that given sufficient sequencing depth the whole genome may be covered with transcripts. A recent study that sequenced total RNA from human brain and liver supports van Bakel *et al*.’s suggestion that unannotated transcripts within introns represent unspliced introns rather than unique independent transcriptional units [106]. And yet another study found that sequenced reads observed in conventional RNA sequencing data sets, previously dismissed as noise, are in fact indicative of unassembled rare transcripts [107]. Therefore the debate about the pervasiveness of transcription continues, but as van Bakel *et al*., and others [30,108] point out, it is time to stop arguing over the content of the transcriptome, and focus on finding evidence for dark matter functions. 

## 5. Discussion and Conclusions

The unprecedented depth of sequence coverage achieved by RNA-seq has revealed how much of the human transcriptome is still uncharacterized. Many novel transcripts are still being discovered, stimulating the debate as to the extent to which the genome is transcribed. Non-coding RNAs represent the majority of the human transcripts, and there is no doubt that many of them, initially considered to be transcriptional artifacts, are in fact functional. They play important roles in transcriptional and post-transcriptional gene regulation via both *cis-* and *trans-*acting mechanisms, chromatin modification, control of transcription factor binding, regulation of alternative splicing. These functions have important consequences for development and for diseases, including cancer [30,36,109,110].

Despite current intense research efforts, many of the novel transcripts identified thus far have an unknown function. Most of them have been found only in specific cell types, tissues, or developmental stages [37,100,111]. They lack functional ORFs, have lower expression levels, and are only modestly conserved, although conservation is only a week indicator of functionality [96,112,113]. Occasionally, entirely novel protein-coding genes with strong mRNA expression have been identified [114], but most unannotated transcripts that are protein-coding are alternatively spliced isoforms of known mRNAs [41]. However, as of today the vast majority of alternatively spliced transcripts lack described functions, and the role of alternative splicing itself in gene evolution remains largely unexplored [46]. 

Is low RNA polymerase fidelity the principal cause of the widespread transcription observed in the human genome? We do not have a definite answer to this question [115]. A focus on deciphering the biological functions of transcribed genomic sequences might provide us with a clearer picture. Over the last decade, the estimated proportion of the human genome that might be functional has been constantly adjusted upwards, and today it lies between 10% and 15% [116]. This estimate is still much lower than the ~93% estimate for the transcribed fraction of the genome [96]. In a 2009 review, Ponting *et al.*, argue that a large, but as yet unknown, number of noncoding RNAs cannot be explained solely as the product of transcriptional noise [30]. If ncRNAs were simply transcriptional noise, than their expression levels would not show the wide diversity that is often observed among different tissues. In addition, their nucleotide substitution rates would be very similar to neutrally evolving sequences. Instead, several evolutionary studies suggest that many ncRNAs exhibit signatures of functionality that are more usually associated with protein-coding genes [47,117], or that their low sequence conservation is due to the fact that they are frequently acted upon by positive selection [118,119]. Nevertheless, some percentage of the transcripts observed are very likely the result either of transcriptional noise [120] or of genomic DNA contamination [121]. Even if not functional themselves, these unannotated transcripts might reflect transcriptional processes that facilitate the expression of other genes. Until we can functionally validate these transcripts or gain a better understanding of the range of transcriptional mechanisms involved, the question of how much of the human genome is transcribed will remain an open question. 
